# Big data in IBD: big progress for clinical practice

**DOI:** 10.1136/gutjnl-2019-320065

**Published:** 2020-02-28

**Authors:** Nasim Sadat Seyed Tabib, Matthew Madgwick, Padhmanand Sudhakar, Bram Verstockt, Tamas Korcsmaros, Séverine Vermeire

**Affiliations:** 1 Department of Chronic Diseases, Metabolism and Ageing, TARGID, KU Leuven, Leuven, Belgium; 2 Organisms and Ecosystems, Earlham Institute, Norwich, UK; 3 Gut microbes in health and disease, Quadram Institute Bioscience, Norwich, UK; 4 Translational Research in GastroIntestinal Disorders, KU Leuven, Leuven, Belgium; 5 Department of Gastroenterology and Hepatology, KU Leuven University Hospitals Leuven, Leuven, Belgium

**Keywords:** IBD, ulcerative colitis, Crohn's disease

## Abstract

IBD is a complex multifactorial inflammatory disease of the gut driven by extrinsic and intrinsic factors, including host genetics, the immune system, environmental factors and the gut microbiome. Technological advancements such as next-generation sequencing, high-throughput omics data generation and molecular networks have catalysed IBD research. The advent of artificial intelligence, in particular, machine learning, and systems biology has opened the avenue for the efficient integration and interpretation of big datasets for discovering clinically translatable knowledge. In this narrative review, we discuss how big data integration and machine learning have been applied to translational IBD research. Approaches such as machine learning may enable patient stratification, prediction of disease progression and therapy responses for fine-tuning treatment options with positive impacts on cost, health and safety. We also outline the challenges and opportunities presented by machine learning and big data in clinical IBD research.

## Introduction

Precision medicine holds great promise to improve the landscape of IBD course of care for an individual patient, providing the most beneficial therapy while minimising the risk. The ultimate goals of precision medicine include stratifying patients based on disease subtypes and severity, disease progression and treatment response using personal and clinical data coupled with molecular profiling data of patients.^1 2^ IBD, with its two main subtypes, Crohn’s disease (CD) and UC, is a complex inflammatory disease with a wide range of contributing factors including host genetics, immune system, environmental exposures and the gut microbiome.^3–5^ The inherent complexity of the disease introduces a large number of confounding factors, which stand in the way of accurate diagnosis and precision medicine.^6^

**Figure 1:**
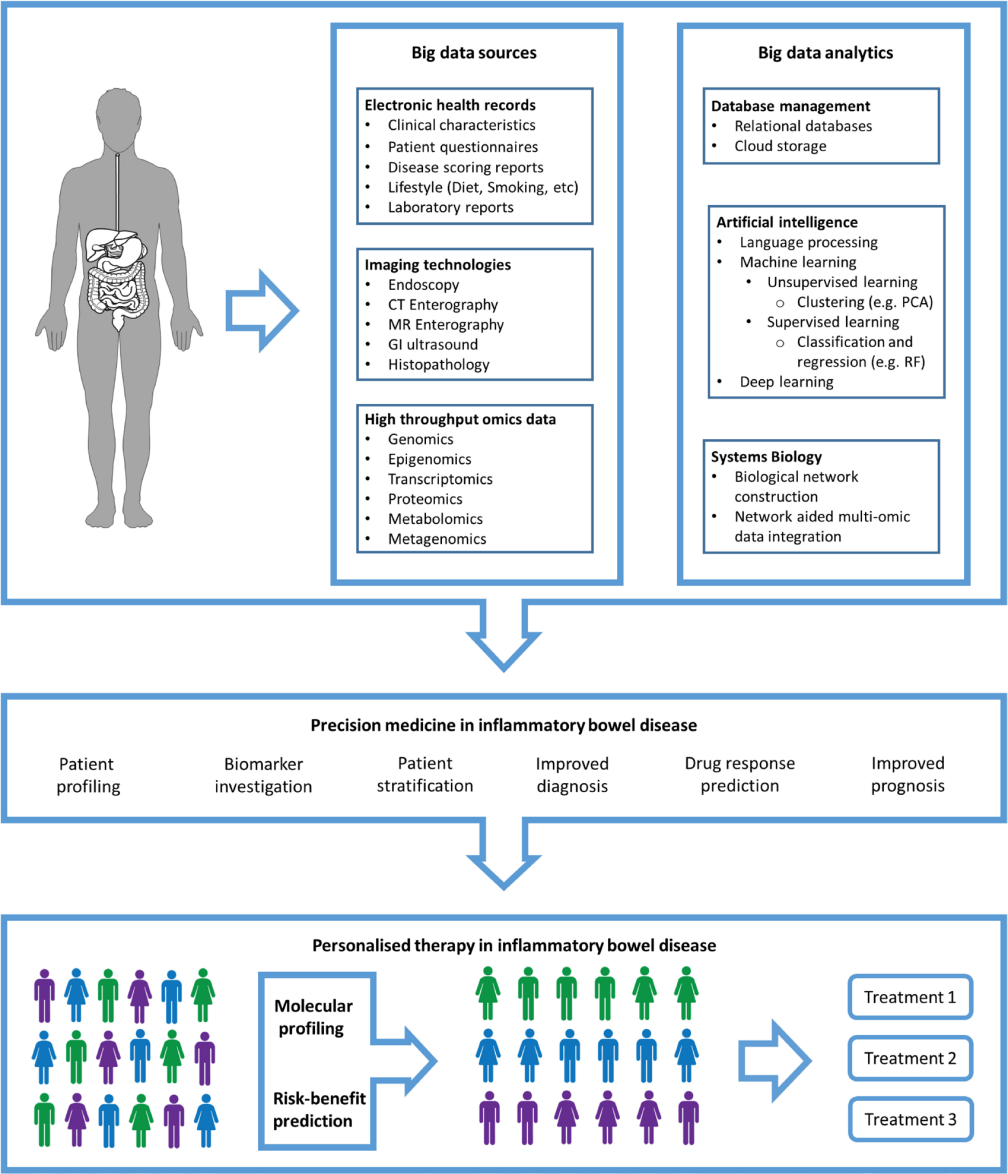
Precision medicine in IBD. Generation of big data from thousands of individuals, along with analytical advancements such as machine learning and systems biology, assists the application of precision medicine and therefore allows patient stratification for personalised therapeutic intervention and disease management strategies. MR, magnetic resonance; PCA, principal component analysis; RF, random forest.

The term ‘big data’ is generally referred to as large volume of rapidly produced data from variable sources, known as the three ‘V’s (volume, velocity and variety).^7^ Over the past decades, the production and availability of data that could inform healthcare has increased remarkably mainly due to technological advancements and falling costs of data generation. Most important sources of data in IBD comprise study cohorts, clinical trials, administrative and electronic health record databases, patient-reported outcomes databases, medical imaging databases and omics datasets (including genomics, transcriptomics, proteomics and metabolomics, as well as environmental omics) (figure 1). The use of big data in IBD allows medical researchers to reveal disease-related trends, associations and patterns to propel our understanding of IBD forward and to inform clinical practice.^2 8^ However, due to the high complexity of big data and the long list of confounding factors, interpreting these data is not trivial and warrants approaches that can uncover hidden patterns in these large and complex datasets.^9^

Recent developments in computational biology have driven the integration of big data and molecular networks using the principles of systems biology and machine learning. Systems biology centres around the holistic and mathematical modelling of complex biological system.^10^ Machine learning is a subset of artificial intelligence, which refers to the ability of algorithms to learn from data in order to detect patterns and make decisions (without explicitly being programmed what to do) (Box 1).^11^ Machine learning algorithms provide the means and opportunity to investigate large amounts of data and thus help identify patterns behind complex medical conditions. These analytical approaches allow categorisation of patients based on their specific differences through screening a patient’s genome, transcriptome, proteome, epigenome, immunome and microbiome. Integrating the omics datasets using systems biology-based approaches may advance understanding of the underlying causative factors in individual patients. The arrival of systems biology and machine learning into IBD clinical research has allowed researchers to capture complex associations andincreased understanding of disease mechanisms in IBD. In this narrative review, we provide an overview of the sources of big data in IBD. We discuss how artificial intelligence could help us better understand IBD pathogenesis and how some components of it have already begun to shape our knowledge of IBD. We address how artificial intelligence could contribute to the diagnosis and prognosis of IBD, and whether it could assist with predictions of therapy efficacy and adverse effects. As a final point, we argue the potential that artificial intelligence provides for personalised medicine in IBD and evaluate the feasibility of big data in IBD disease management.

Box 1 **Artificial intelligence terminology**

Artificial intelligence: the field of computer science which concerns the theory and development of computers to perform tasks which usually requires human intelligence, such as imagine classification, speech recognition and decision-making.

Machine learning: a field of artificial intelligence which refers to the computers’ ability to learn to make decisions or detect patterns (without explicitly being programmed) from data.

Deep learning: a subfield of machine learning that exploits many layers of non-linear information processing for supervised or unsupervised feature extraction and transformation, and for pattern analysis and classification using various neural network frameworks.

Supervised learning: the task of an algorithm learning a function that maps an input to an output based on provided example data.

Unsupervised learning: the task of a machine learning algorithm to learn the underlying data structure of unlabeled example data, for example, finding commonalities, leading to insights and therefore a greater understanding of the example data.

Classification: the process of predicting a class/subcategory of given data points from known example data.

Generalisation: refers to how well the machine learning model learns the underlying data and the model’s ability to apply this to specific examples not seen by the model during training.

Ensemble learning: the union of homologous or heterogeneous machine learning algorithms whose predictions are combined to achieve greater performance than just the individual machine learning algorithm could achieve alone.

Support vector machine: this is a discriminative classifier which determines classes from a separating hyperplane. Through the use of a kernel, SVMs can be adapted to suit non-linear problems.

Random forests: a homologous ensemble algorithm which constructs a great number of decision trees at training.

Matrix factorisation: an algorithm which extracts meaningful association from an incomplete data matrix and transforms them in a lower dimensional latent space, also known as recommender systems.

## Role of machine learning and systems biology in the interpretation of big data in IBD research

The main challenge faced by many scientists is to extract meaningful information through integrating different sources of data and thereby discover disease association patterns. Classical statistical methods are not powerful enough to explain the underlying milieu of pathogenic and causative factors in IBD. Hence, scientists have adopted different analytical methodologies. Generally, such analytical methodologies are categorised into two main groups, namely, systems biology and machine learning. These are more powerful and flexible methods in biomedical data science and have the potential to uncover novel insights into disease pathogenesis.^12 13^

Systems biology paves the way for data integration and analysis from a functional perspective, and it has assisted in identifying the pathophysiological mechanisms of IBD. The approach of systems biology typically involves the use of networks (mostly molecular networks such as protein–protein interaction networks, regulatory networks involving transcription factors and metabolic networks) to capture the physical and signalling interactions and to interpret contextual measurements such as expression of genes, proteins and metabolites. This approach thereby provides a framework to identify key components and/or pathways which mediate the pathogenesis of the disease. Brooks *et al* identified different clusters of patients with UC using network footprints created by combining mutation data, protein–protein interaction networks and gene expression data.^14^

In the past decade, machine learning has attracted much attention from groups engaged in IBD research, owing to its ability to learn complex patterns and make prediction. With machine learning as a framework, several attempts have been made to use different types of omics and clinical datasets to improve our understanding of disease mechanism. Given that omics datasets, such as RNAseq data, comprise expression information of thousands of genes (features) with far few samples, feature selection is of great importance. Machine learning algorithms take advantage of data-dependent automatic feature learning, while systems biology approaches need to be manually programmed. Machine learning algorithms can learn how to integrate several predictors to identify a representative subset of input data^15^; for example, a machine learning algorithm using the concept of random forests identified a panel of 50 faecal bacteria capable of distinguishing active and remission states in patients with CD.^16^

### Genomics

IBD is considered as a polygenic disease, with the exception of rare monogenic cases.^17^ The notable example of research into the genetic basis of IBD is the introduction of *NOD2* as the first CD susceptibility gene.^18^ To date, the continued search for genetic determinants of IBD identified 242 variants associated with IBD,^17^ of which 45 have been fine mapped to statistically significant causal variants. Interestingly, associated regions indicate that there is a profound overlap between IBD and other immune-mediated inflammatory diseases. However, merely a small percentage of heritability is explained by the identified loci.^17^

To further resolve the genetic architecture of IBD, machine learning and data integration could be employed to propel the gene discoveries. The main issue with association studies is the imbalance between the number of patient samples and the number of single-nucleotide polymorphisms (SNPs) that are being analysed. In addition, the classical genotype–phenotype association at high statistical confidence neglects a considerable fraction of genetic variation. Machine learning could be used to detect meaningful patterns containing thousands of DNA variants, regardless of the statistical significance level.^19–22^ This could result in predictions of genetic markers and variants with greater accuracy. An exemplary study was conducted using data from the International IBD Genetics Consortium’s Immunochip project. To reduce the number of SNPs, Wei and colleagues applied a less rigid statistical confidence limit (p values of <10^4^ and minor allele frequency of <0.01) followed by a machine learning classifier-based feature selection method (the penalised logistic regression model). The authors defined 573 SNP-based CD and 366 SNP-based UC predictive models with superior area under the receiver operating characteristic curve (AUC) values than the log OR-based models (AUCs of 0.86 (95% CI 0.85 to 0.86) and 0.826 (95% CI 0.81 to 0.83) for CD and UC, respectively).^23^ Another interesting study was conducted using the UK Inflammatory Bowel Disease Genetics Consortium and UK10K consortium for the controls, which cumulatively comprises approximately 8000 individuals (4280 patients and 3652 controls). In this study, a machine learning model, a support vector machine (SVM), was used to hunt for novel genetic variants, which resulted in the identification of a missense variant in *ADCY7* associated with UC with a frequency of 0.6%.^24^ A recent study reanalysed the Immunochip dataset using different machine learning models, including random forests and neural networks. Romagnoni *et al* identified new variants with minor effects, in addition to almost all of the previously known variants among the best predictors of CD.^25^

Advancements in sequencing technologies allow a more in-depth genomic screening. Scientists have used whole genome/exome sequencing particularly to discover rare genetic variants, such as *NOX1*, contributing to very early-onset IBD.^26^ Machine learning methodologies, particularly deep learning, are resourceful tools for not only making predictions but also extracting biomedical insights.^27–30^ In a notable publication, Zou *et al* provided a primer on deep learning for genomic data analysis accompanied with practical guidelines for the discovery of DNA-binding motifs.^31^

### Transcriptomics and proteomics

Investigating the downstream effects of genomic aberrations, namely, on the transcriptome and proteome, provides additional molecular details to unravelling IBD pathogenesis. Differential gene expression analysis has been used to identify key genes and pathways underlying IBD pathogenesis. Transcriptomic analyses of human ileum and colonic samples have helped to uncover the roles of different pathways driving inflammation in IBD. For example, inflamed and non-inflamed tissues have altered gene expression in CD and UC. To investigate the functional significance of these modifications and to characterise their molecular signatures in colonic tissue, an integrated systems approach has highlighted significant enrichment in proteasome and apoptosis pathways.^32^ With protein–protein interaction network analysis, Li *et al* identified *MAPK3*, *NDRG1* and *HLA-DRA* as key players in disease pathogenesis. Following a similar approach, Hong *et al* identified altered gene expression profiles and key cellular pathways in patients with inflamed and non-inflamed intestinal mucosa with CD, including immune response, chemokine signalling and cell adhesion.^33^

Weighted gene coexpression network analysis allows researchers to detect genes that are upregulated or downregulated in tandem under specific conditions.^34 35^ For example, Lin *et al* revealed important pathogenic roles for *IL-8* and *MMP-9* in the colonic tissues of patients with UC by combining gene coexpression and protein–protein interaction networks.^36^ A similar study in the context of gene expression alteration in different stages of CD by Verstockt *et al* pinpointed that dysregulation of the coexpression network is more evident in newly diagnosed and late-stage CD compared with recurrent CD.^37^ Likewise, this network approach can elucidate biological mechanisms driving treatment resistance to biological therapies, such as with tumour necrosis factor (TNF) inhibitor agents.^38^ Another functional approach to explore the gene expression data is metabolism-level interpretation using Recon 2,^39^ the model of the human metabolic network. Using this model, critical pathways such as cellular transport of thiamine and bile acid metabolism have been identified.^40^

Yuan and colleagues reported 41 discriminatory IBD-related genes by combining machine learning and systems biology. In searching for novel candidate genes, the authors used a two-step feature selection on microarray data from patients with CD, UC and control individuals. First, they ranked thousands of genes according to their correlation to diagnosis and the redundancies between each gene related to all other genes in the ranked list. Then, using an SVM as a machine learning classifier, they identified a feature set containing 21 genes, which yield the highest prediction accuracy. Additionally, based on the concept of functional similarity among closely related proteins, the authors used the protein–protein interaction network of the proteins encoded by those 21 genes and applied the shortest path approach (typically defined as the path with the least number of links between two proteins in a network) to find an additional 20 candidate genes.^41^ In another interesting study by Isakov *et al*, novel candidate genes were identified by developing a machine learning model trained on expression values of known IBD susceptibility genes and their functional annotations. The authors used the feature importance of a machine learning classifier as the feature selection method.^42^

### Environmental ‘omics’

The gut microbiota, which comprise intestinal bacteria, fungi, archaea and viruses, is an essential part of the human GI tract and plays a pivotal role in human health. In homeostatic condition, there is a state of immunological tolerance to the commensal intestinal microbiota. It has been established that perturbation of composition, function and structure of the gut microbiota, known as dysbiosis, is one of the key players in IBD pathogenesis.^43^ However, it is still not clear whether this dysbiosis is the cause or consequence in patients with IBD.

There is a decline in both species diversity and richness in patients with IBD. Several studies have reported an increase in the abundance of certain species from the Proteobacteria phylum, such as *Escherichia coli*, and a decline in anti-inflammatory butyrate-producing bacteria species, such as *Faecalibacterium prausnitzii*, belonging to the Firmicutes phylum. Additionally, a longitudinal study suggested an increase in dynamic fluctuation of the gut microbiome composition in patients with IBD.^44^

Much less is currently known on the role played by viruses in the dysbiotic state in patients with IBD. Recent advances in sequencing technologies and data analytic techniques have enabled in‐depth characterisation of microbiota communities to investigate IBD pathogenesis using meta-level omics datasets, namely, metagenomics, metatranscriptomics, metaproteomics and metabolomics. Deep metagenomics paved the way to study gut resident fungi, archaea and viruses in both healthy and disease states. Different stool virome profiles have been observed in patients with IBD compared with healthy individuals.^45^ Zuo and colleagues used machine learning-based clustering to define viral metacommunities in rectal mucosa derived from patients with UC. The predominant viral community among patients with UC showed decreased viral diversity, richness and evenness, particularly among *Caudovirales* species. However, two species of *Caudovirales* (*Escherichia *phage** and *Enterobacteria phage*) were much more common among patients with UC compared with healthy controls. This suggests a loss of corelationship between the viruses and bacteria, which can cause microbiota dysbiosis and intestinal inflammation.^46^

The interplay between the microbial composition and metabolism of the gut is an interesting nexus in IBD. While much of the previous research on this interaction level has been interpretive in nature, most of the studies on the gut protein and metabolic composition used shotgun metagenomic technique. Thus, by comparing the abundance of enzymatic genes across samples, scientists have been able to infer the effect of variations in microbial composition on the protein and metabolic levels. An example of this is the study by Greenblum *et al* in which they used faecal metagenomics to build metabolic networks. They demonstrate topological differences by which IBD-associated metabolic networks interact with the gut environment and the host.^47^ There is a growing number of investigations applying the approaches of metaproteomics and metabolomics. Particularly, there are two avenues in which metaproteomics-based investigations have been employed, the mucosal–luminal interface analysis and the stool metaproteome profiling. Li *et al* investigated the protein co-occurrence network at the mucosal surface of six different colonic regions. Employing weighted correlation network analysis and multiple clustering methods such as hierarchical clustering, they identified distinct functional protein modules (protein clusters that alter together) in association with non-IBD, UC and CD disease states.^48^ In addition to systems biology methods, machine learning could be applied to define relevant protein clusters. Profiling of stool samples revealed that metaproteomic signatures in patients with CD differ from those of healthy individuals. By integrating metagenomics and metaproteomics, and applying a hierarchical clustering method, Erickson *et al* reported a depletion of several microbial proteins in patients with CD with ileal involvement, such as proteins in the butyrate pathway which corresponded to a reduction in the Firmicutes phylum.^49^

### Multiomics data integration

In more recent investigations, researchers have been collecting different levels of omics data from patients with IBD to investigate the crosstalk between the key players in IBD pathogenesis. An interesting area in which multiomics data integration has been applied is to characterise the dysregulated multifaceted interactions between various host and microbial factors in IBD. For example, Häsler *et al* studied the transition of intestinal homeostasis to dysbiosis by integrating multiple levels of data, namely, the mucosal transcriptomic, post-transcriptional alterations and the mucosal microbiome of patients with UC and CD in comparison with healthy individuals. The authors identified the enrichment of host transcript splicing events as a result of the interplay between microbial and host factors which probably mediate the transition of intestinal homeostasis to dysbiosis in patients with IBD.^50^ In order to investigate the dysbiosis at the functional level, Lloyd-Price *et al* followed up 132 patients with IBD for 1 year and performed extensive molecular profiling of all patients. The authors revealed a distinctive upsurge in the ratio of facultative anaerobes to obligate anaerobes, along with disruptions at the molecular level, including microbial transcription division (within clostridia) and metabolite disruptions (acylcarnitines, bile acids, and short-chain fatty acids). Additionally, they reported noticeable alterations in the composition and function of microbiota with regard to different disease activity states.^51^

## Current paradigm of IBD disease management and its limitations

The scope of IBD treatment is extending swiftly, with the introduction of new biologics and small molecules as a result of the improved understanding of the disease pathophysiology. With novel treatment options (targeting different aspects of IBD pathophysiology) such as anticytokine or chemokine agents, antiadhesion molecules, stem cell therapy and manipulation of the gut microbiota becoming increasingly available, it is time to move beyond the ‘one-size-fits-all’ approach.^52^

**Figure 2:**
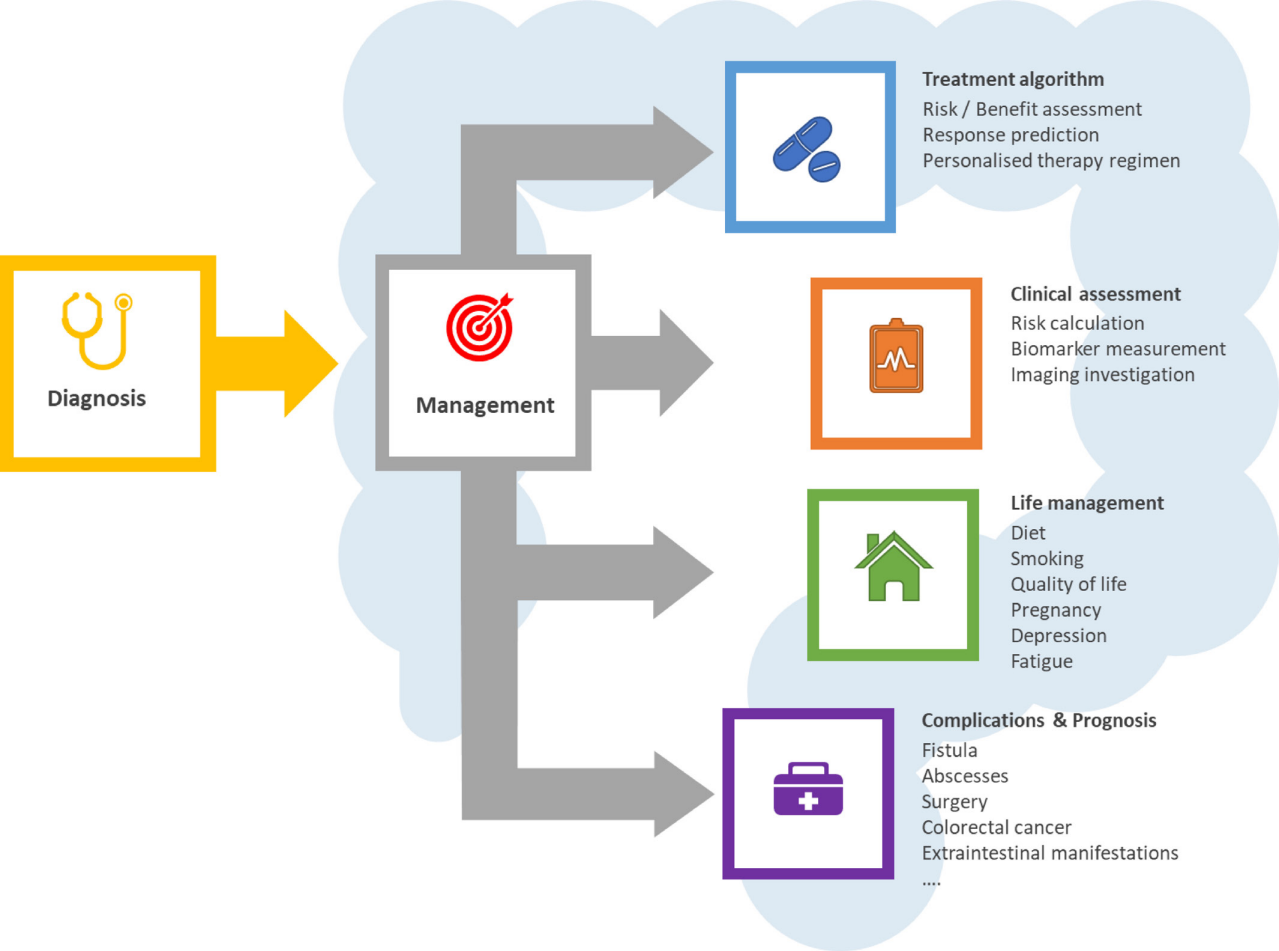
Clinical management of IBD from the point of diagnosis to life-term monitoring and follow-up. Each stage of the disease management process can potentially be subjected to precision medicine-aided improvement of patient care to reduce the socioeconomic burden on patients, clinicians and the healthcare system.

IBD management (figure 2) encompasses three different stages, starting with diagnosis, followed by the assessment of disease and the choice of therapy regimens, follow-up assessments and associated treatment changes, if necessary. Disease monitoring is key and is currently carried out by tracking different markers like faecal calprotectin, serum C reactive protein, also colonoscopy and/or medical imaging technologies such as abdominal ultrasound and MRI.^53 54^ Hitherto, the clinical decision on the choice of therapeutic strategy depended on the response and tolerability of treatment in patients. However, in light of recent innovative therapies in IBD, a more accurate method is warranted to assist and complement existing management.^55^ In recent years, there has been an increasing interest in the application of machine learning in IBD clinical research. Using machine learning for personalised predictions will not only strengthen medical care and improve outcomes but also considerably decrease healthcare expenditure. Despite the importance of health economics, there are little published data on the cost-effectiveness of artificial intelligence in healthcare. An interesting example is the study conducted by Bremer *et al*, who deployed a machine learning methodology to predict the individual outcome and costs for patients with depressive disorders prior to the start of intervention in order to allocate patients to the most beneficial treatment.^56^ In the field of gynaecology, Wang *et al* proposed a machine learning-based strategy for urinalysis which significantly increased the detection rate of the pathogen *Trichomonas vaginalis* in a cost-effective manner.^57^

### Diagnosis and risk stratification

The current paradigm of IBD classification, which relies on invasive ileocolonoscopy and biopsies, does not adequately capture the broad spectrum of phenotypes of the disease or the patient-specific manifestations of its comorbid conditions. Recent research has focused on identifying and evaluating potential non-invasive diagnostic markers to diagnose IBD, differentiate it from other disorders and potentially improve its classification. There is great interest in the diagnostic value of genomics data, with over 240 IBD-associated risk loci already identified using genome wide association study (GWAS) data. A genotype–phenotype study associated three loci, *NOD2*, *MHC* and *MST*, with subphenotypes of IBD, particularly disease location.^58^ Exome sequencing has arisen with the promise of unravelling the genetics of complex diseases. However, extracting disease-associated sequence variants is challenging due to inherited diversity of genomic variation. By incorporating exome sequence data with biological knowledge, such as functional interaction networks, into a matrix factorisation-based machine learning model, Jeong and Kim were able to distinguish patients with CD from healthy individuals (AUC=0.81).^59^

Likewise, molecular and cellular signatures can enable stratification of patients based on underlying pathways that drive their disease. Gene expression profiling is a major area of interest in the search of clinically associated signatures for IBD class prediction. To identify a set of genes distinguishing between UC and CD, novel machine learning-based methods have been used. Two examples which stand out are the PROPhet software package,^60^ which automatically selects the best classifier and the optimal selection of genes to distinguish disease subtypes. Montero-Meléndez *et al* used this technique with microarray gene expression profiling of colonic biopsies to identify predictive transcriptional signatures associated with either CD or UC.^61^ The second example is the Probabilistic Pathway Score, which is a pathway-based machine learning model that uses gene interactions to identify molecular pathways affected by the disease of interest and identify similarities and differences between them.^62^ Proteomic signature is another promising nexus in biomarker research. Machine learning models have also been used with proteomic data to stratify patients with IBD. For example, Seeley *et al* investigated the protein signatures from colonic tissues using an SVM machine learning classifier trained on 25 peaks from histology-based mass spectrometry data. The model was able to discriminate patients with CD and UC from each other with an accuracy rate of 76.9%.^63^ Another interesting area of biomarker research in IBD is microRNAs (miRNAs), a group of small noncoding RNA molecules which control gene expression and protein production and are detectable in many sources such as blood and urine. Hence, miRNAs hold great promise as potential non-invasive diagnostic markers. miRNAs are dysregulated in IBD.^64^ Therefore, researchers have attempted to demonstrate the diagnostic value of circulating miRNAs signatures in the blood as diagnostic biomarkers using machine learning modelling, including random forests and SVM.^65 66^

An interesting example of exploring the diagnostic value of a set of biomarkers is the study conducted by Plevy *et al* combining genetic variants, serological and inflammatory markers to establish a diagnostic model to distinguish patients with IBD from those without IBD (healthy individuals or other diseases) and to separate patients with CD from UC. Based on the data from 1520 individuals, the authors selected 17 statistically significant markers and trained a random forest classifier, a machine learning algorithm, to differentiate the clinical groups.^67^

Machine learning approaches also hold great promise in unravelling disease-specific microbial signatures. Multiple machine learning-based microbiome frameworks have been established such as Multivariate Association with Linear Models (MaAsLin),^68^ Metagenomic prediction Analysis based on Machine Learning^69^ and phylogenetic convolutional neural networks^70^ which incorporate patient clinical data, knowledge of microbial strains and knowledge of phylogenetic structure, respectively. Integrating additional information is expected to enhance the classification performance of microbiome-based machine learning models. As an example, Gevers *et al* were able to use rectal mucosa-associated microbiome signatures to distinguish paediatric patients with CD from patients with other GI tract conditions by integrating patient clinical data age, gender and past antibiotic use with the microbiome profiles using MaAsLin workflow.^71^

**Figure 3:**
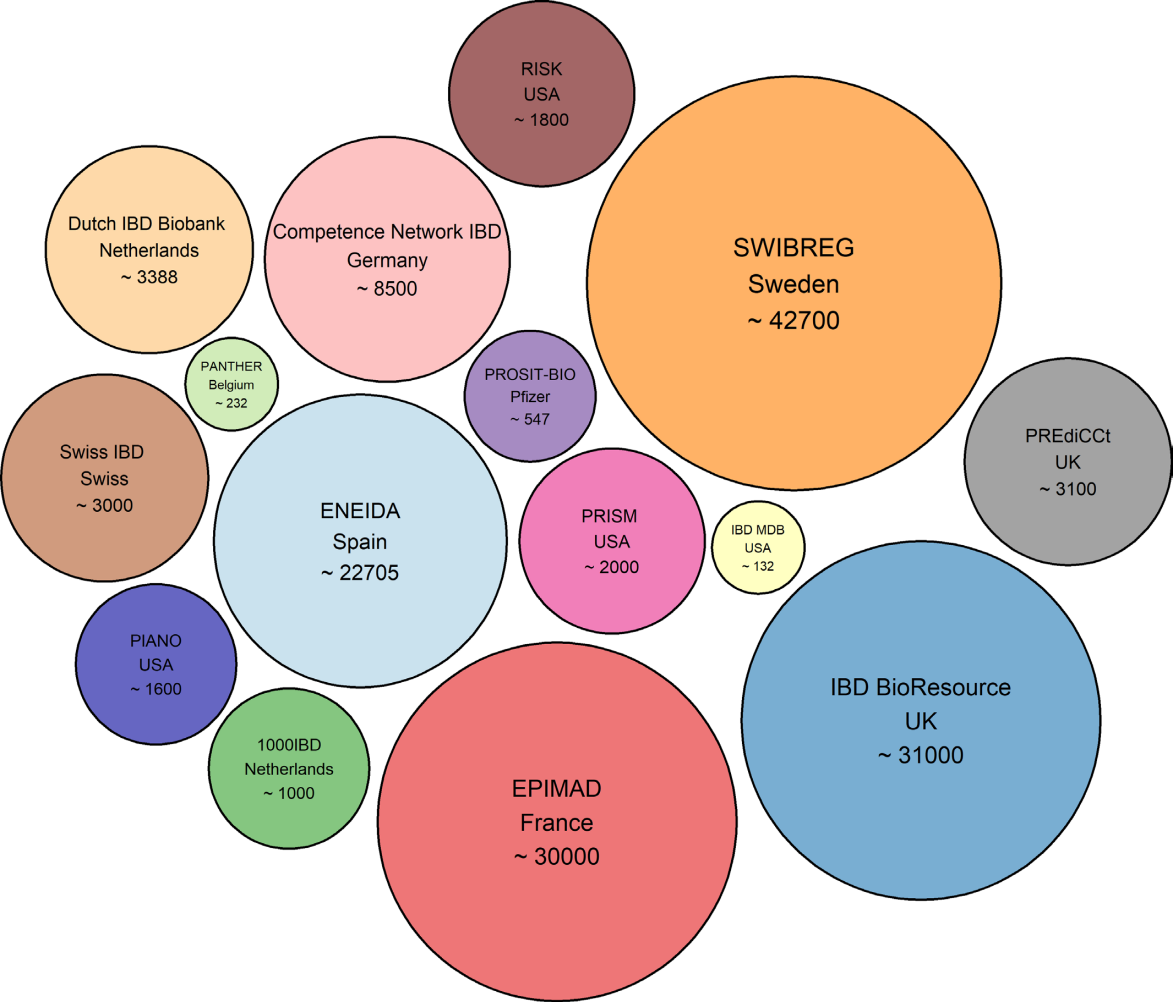
Academic initiatives with cohorts/biobanks in IBD. The numbers in each circle represent the approximate patient cohort size.

While the initial results of biomarker identification are promising, there is still a long way to go before these biomarkers can be applied in clinical practice, mainly due to the heterogeneity of the disease, diverse comorbidity factors and, importantly, lack of validation. The emergence of big data and big data analytics has led to a pile of studies and hypotheses. Although these approaches show great potential in a study-by-study basis, to translate these findings to a clinical setting, it is crucial to distinguish true discoveries from red herrings. Therefore, replication and validation studies in much larger cohort sizes are required. To achieve this, large and up-to-date clinical biobanks with a variety of different data types, including molecular, clinical and host characteristics, will be required to fully leverage these analytical methodologies. In precision medicine era, many national and international collaborative efforts are under way aimed at improving clinical research (figure 3).^72–84^

### Advances in imaging technologies

Image recognition is one of the major applications of artificial intelligence, particularly deep learning, and holds great promise in assisting the fields of biological and medical imaging. Deep learning is a collection of algorithms in the field of machine learning with an outstanding ability to decode the contents of images. This has led to a proliferation of studies with an attempt to automate the interpretation and the evaluation of medical images, such as endoscopy, histopathology, and CT/MRI. Evaluating endoscopic inflammation, characterisation of lesions and assessment of mucosal healing is essential for proper management in IBD. However, endoscopic assessment of inflammation in IBD is highly subjective with high interobserver variability. Computer-aided scores would be much more objective for the interpretation of the endoscopic images.^85^ For example, a deep learning-based model showed performance comparable to those of experienced gastroenterologists for the classification of endoscopic severity of UC into two groups: remission (Mayo 0 or 1 endoscopic score) and moderate to severe (Mayo 2 or 3 endoscopic score).^86^ A novel objective computer-based score to assess UC disease activity based on endoscopic images has been developed. In particular, deep learning has been used to extract different layers of pixel data, such as measuring the redness degree through extraction of the intensity and distribution of red pixels in the red density score in UC.^87 88^ Similarly, assessment of CT/MRI images in IBD is extremely subjective; therefore, computer-aided scores could potentially overcome interobserver variation. A semiautomated image analysis software showed a performance similar to those of experienced radiologists for the assessment of CD structural bowel damage in abdominal CT-enterography data.^89^ Also, machine learning methods and algorithms have been applied to predict the grading of severity of CD in abdominal MRI data.^90 91^ Additionally, machine learning algorithms could assist with the time-consuming assessment of wireless capsule endoscopy data. It paves the way for automated analysis of wireless capsule endoscopy images to detect CD lesions via detection of predefined structural and textural characteristics, as well as enhancement of the underlying pixel information.^92 93^

**Figure 4:**
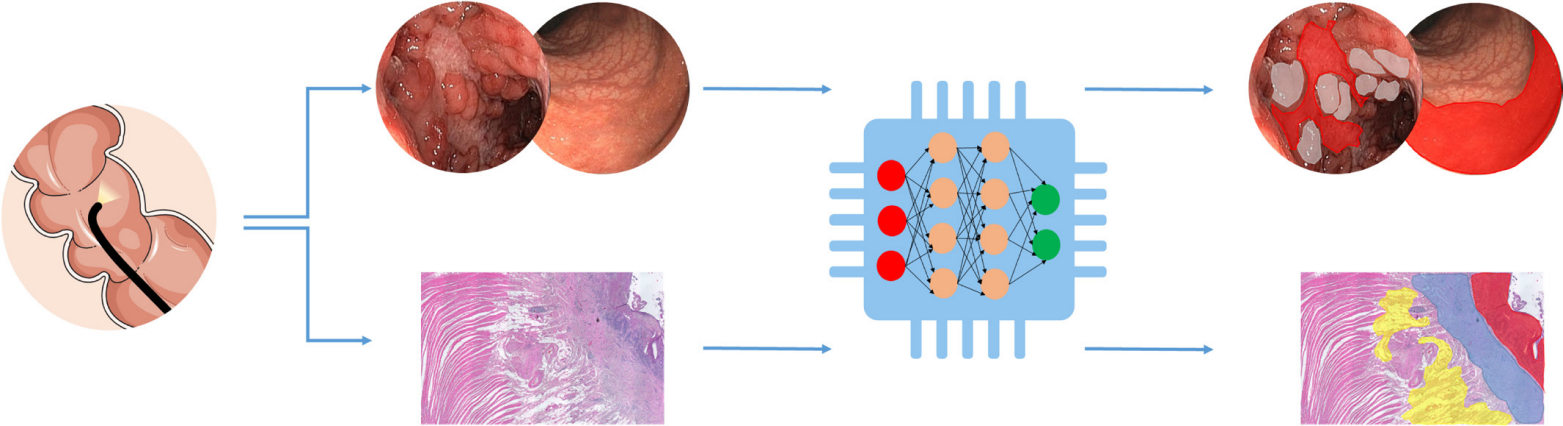
Artificial intelligence in medical imaging. Graphical representation of a simple deep learning-based image segmentation approach to predict boundaries of inflamed areas. The top section of the figure represents the endoscopic image of colonic CD demonstrating the ‘cobblestone’ appearance and ulceration. Using a simple deep learning-based image segmentation method inflamed boundaries could be predicted: cobblestone in grey and inflamed ulcer in red. The bottom section of the figure illustrates a histopathology image of inflamed stenosis from ileal CD. A deep learning-based method could be used for image segmentation and predicting boundaries of inflamed areas: acute infiltration (ulcer) in red, muscolari mucosae thickening in blue and adipocytes hyperplasia in yellow. CD, Crohn’s disease.

Machine learning may also improve the analysis of histopathology and possibly tackle the unmet need of patients with unclassified IBD. Raman microspectroscopy as a cell and tissue diagnostics approach has been investigated to distinguish different IBD subtypes. Bielecki *et al* proposed that a machine learning-based workflow is capable of distinguishing tissue morphology among healthy subjects, CD and UC with great accuracy.^94^ Ultimately, artificial intelligence is promising in medical imaging and will undoubtedly have a considerable impact on endoscopy practice in the future (figure 4).

### Predicting prognosis

Predicting disease progression and severity is pivotal to the design of appropriate disease management strategies for individual patients. Machine learning has the potential to assist with this. Extraction of information from routinely collected electronic medical records (EMRs), such as physician’s clinical observations and endoscopy reports, will allow researchers to perform prognostic research on longitudinal data. A machine learning model trained on codified information (International Classification of Diseases, Ninth Revision (ICD-9)) retrieved from EMRs, including a set of baseline laboratory parameters, patient demographics and clinical characteristics, accurately (AUC= 0.93) predicted disease severity in patients with CD.^95^ Similarly, Waljee *et al* constructed a random forests machine learning model to predict IBD-related hospitalisation and outpatient steroid use, as surrogate markers of disease flares (AUC=0.87, 95% CI 0.87 to 0.88). The authors pointed out older age, high serum albumin, platelet counts, immunosuppressive medication, history of corticosteroid use and hospitalisation as risk predictors.^96^ One way to improve and facilitate data extraction from plain text in medical records is by employing natural language processing (NLP), another field of artificial intelligence.^97^ For example, an NLP-based model showed superior performance in comparison to an ICD-9-based model for extracting extraintestinal manifestation data from EMRs.^98^ In the IBD therapeutic space, Cai *et al* applied NLP to clinical notes in identifying the risk of arthralgia in two groups of IBD patients: one treated with vedolizumab and another with TNF inhibitor.^98^ Hou *et al* examined the performance of NLP-based software to classify the endoscopy procedure in patients with IBD that was performed in a diagnostic or follow-up context by mining the pathology reports.^99^

Most investigation in prognostic research has centred on investigating the diversity of underlying disease pathophysiology aiming to identify predictive correlates, which shed light onto the factors prompting disease progress, severity and clinical manifestations. Genome-wide association studies in patients with CD pinpointed the distinct genetic bases of susceptibility and prognosis and hence separate biology. These prognosis-associated SNPs are enriched for pathways involved in the regulation of innate and adaptive immune responses and responses to microorganisms. Among those, four loci have been identified to be significantly associated with prognosis in CD, namely, *FOXO3*, *XACT*, a region upstream of *IGFBP1* and *MHC*. This serves as the point of departure for better understanding of the biology that determines disease prognosis.^100^ Advancement of omics techniques and data analytics have led to molecular and functional-based disease classification. For example, on combining mucosal gene expression, metagenomics and CD4 +T cell population signatures, Tang *et al* employed a machine learning approach to define a list of 26 predictors, which were effective in distinguishing between normal intestinal regions and those with active inflammation in IBD patients. Using network analysis to further interpret the inferred predictors, the authors pinpointed the role of *SAA1* in the induction of IL17 and IL22 secretion by CD4+ T cells in relation to *Bacteroides* abundance.^101^

To date, various studies have assessed the predictive value of gut microbiota. Machine learning models, especially random forests, are used extensively in microbiome research due to their ease of understanding, excellent performance and incorporated feature selection (via estimating feature importance). Douglas and colleagues studied microbial taxa and their inferred function in intestinal biopsies of 20 treatment-naive paediatric patients with CD and 20 control patients. The authors pointed to the predictive value of microbiome profiling using 16 s rRNA sequencing for the disease state, whereas metagenomic-based identified markers performed best for classifying treatment response.^102^

When large integrated EMRs and multiomics datasets are combined with a powerful and robust machine learning framework, they can achieve exceptional results. Cushing *et al* identified a unique expression profile in anti-TNF-naive and anti-TNF-exposed patients with CD that could predict postoperative disease recurrence. The authors uncovered 30 influential transcripts in anti-TNF-naive patients using random forests-based machine learning models built on demographic and clinical data extracted from the EMR and transcriptomic profile of non-inflamed ileal tissue.^103^

These methodologies provide a promising initiative to the application of machine learning to predict IBD disease course and outcome, a research scope demanding comprehensive and longitudinal investigations. By expansion of data resources as well as advancement in analytic approaches, prediction of prognosis and identifying low-risk and high-risk patients doubtlessly become feasible. Future studies should aim at mining health records and integrating them with multiomics data.

### Predicting drug response

In the past decades, enormous efforts have been made to predict the response to medications. Since prospective indicators of drug responses are expected to have a big impact on pharmacoeconomics, machine learning approaches have been applied to dissect the underlying complexities and predict responses to drugs used in IBD treatments. Integration of clinical and laboratory data has been used for monitoring drugs with narrow therapeutic window, such as thiopurine, to assess the risk of developing adverse events. Currently, evaluation of clinical efficacy and risk management of thiopurine is either through blood count or measuring and monitoring of the level of its metabolites 6-thioguaninenucleotide, as an indicator of response, and 6-methylmercaptopurine, which is associated with the risk of hepatotoxicity. Waljee and colleagues studied the predictive value of a set of clinical and laboratory data to differentiate clinical responders from non-responders using a machine learning model, random forests. The proposed model has an AUC of 0.85, in contrast to the conventional model with an AUC of 0.59.^104^ Subsequent work has shown significant clinical benefits, including decreased steroid prescriptions, hospitalisations and surgeries.^105^

Using clinical trial data from the GEMINI I and GEMINI II studies with vedolizumab, Waljee and colleagues developed a machine learning model, random forests, incorporating demographic data, clinical data and laboratory tests to predict the likelihood of achieving week 52 corticosteroid-free endoscopic remission in patients with UC^106^ and CD^107^ treated with vedolizumab. Interestingly, the strongest positive prognostic markers in patients with UC were low levels of faecal calprotectin and albumin; and those in patients with CD were low levels of serum C reactive protein and albumin.

An example of efforts to generate and integrate molecular and clinical data to guide treatment relates to identifying biomarkers predictive of drug response. In an interesting study, Zarringhalam and colleagues searched for predictive biomarkers for response to infliximab for refractory UC. First, an in-house algorithm incorporating causal prior knowledge (relationships between genes defined from the literature) with gene expression data was used to define upstream gene regulators. The newly defined features were subsequently used in a machine learning model (panelised logistic regression) to predict patient’s response to infliximab (accuracy=70%). The authors pinpointed interferon gamma (IFNG), lipopolysaccharide (LPS) and TNF as key regulators. They inferred that the lack of response could be due to higher expression of the TNF pathway components, enzymatic dysregulation in the IFNG pathway and activation of the LPS–TLR4 pathway triggered by the presence of Gram-negative bacteria.^108^

Given that the human gut hosts billions of microorganisms, the gut microbiome is increasingly known to be a contributor of drug efficacy.^109^ Doherty and colleagues used a machine learning model using the concept of random forests to predict the therapeutic response to ustekinumab in patients with CD.^110^ The model helped in the identification of microbial signatures such as altered levels of *Faecalibacterium* that were predictive of remission. Similarly, Shaw *et al* performed an analysis using a similar classifier model based on longitudinal microbiome data derived from 19 treatment-naive paediatric individuals diagnosed with IBD and exposed to biologics.^111^ The authors were able to achieve a 76.5% accuracy in predicting responders based on the pretreatment microbiome. These studies suggest that stratification of patients according to their molecular and clinical characteristics would be beneficial for evaluating therapeutic efficacy. Multiomics data integration could prove useful in biomarker discovery for treatment response. Recently, our group identified 10-feature transcriptomic (accuracy of 98%) and 15-feature genomic (accuracy 96.6%) panels predicting endoscopic response to ustekinumab by incorporating genomics and transcriptomics data into a matrix factorisation-based machine learning model in patients with CD.^112^

## Key challenges and opportunities

**Figure 5:**
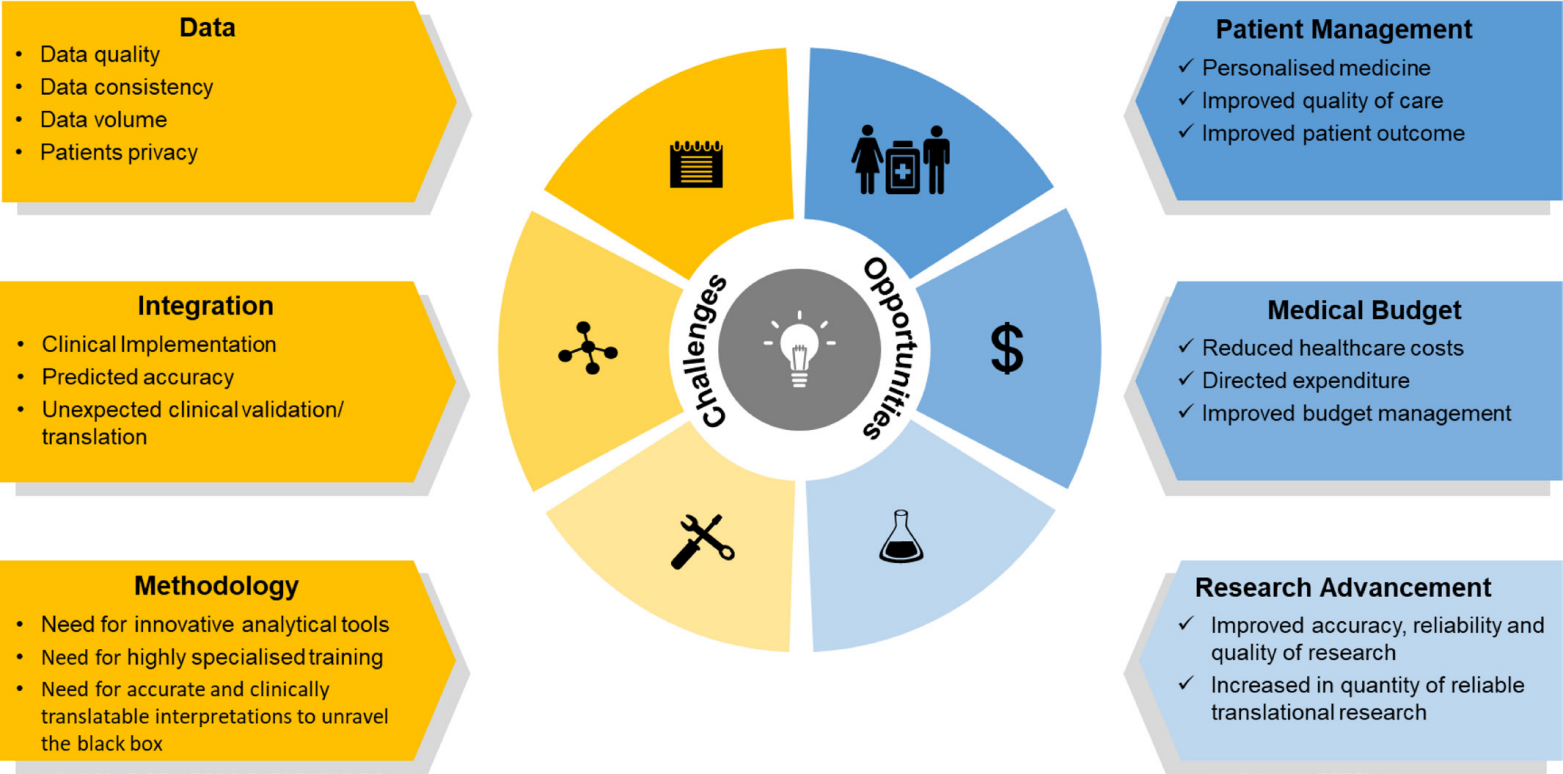
Opportunities and challenges in the use of machine learning and data integration to achieve improved and personalised healthcare in IBD. While challenges exist in generating good quality data in a standardised manner and at a volume deemed suitable for ensuring baseline performance of machine learning models, there remain difficulties in terms of the expertise needed to identify and employ appropriate tools for data integration and interpretation. However, with emerging advances in the data integration field, the incentives and opportunities to advance precision medicine with clinical implications are expected to drive integrative IBD research forward.

Big data and artificial intelligence represent a great step forward in precision medicine with a high reward stand-off. With the potential to simultaneously discover new therapies, make informed treatment decisions and identify disease subgroups, there is a massive effort towards making artificial intelligence commonplace in clinical and biomedical research. The increasing availability of big data, especially multiomics datasets from large IBD cohorts, development of machine learning-based algorithms and systems biology-based tools have enabled the discovery of biological knowledge relevant to IBD. However, key challenges remain especially in the realm of how such datasets become useful in clinical translation and precision medicine (figure 5). Even though existing datasets have yielded interesting biological insights, the number of cases of such datasets resulting in direct clinical benefits, has been few and far in between. This is striking especially given the fact that there is a call for personalised therapies.

This translational gap is not unsurprising since the causality axis for IBD has not yet been established. In part, this could be attributed to the temporal nature (cross sectional or longitudinal) and/or the composition (type of multiomics data types) of datasets. Longitudinal profiling of multiomics datasets even from smaller cohorts may have higher performance and information richness than larger cohorts without longitudinal profiling. This has been demonstrated in other complex diseases such as diabetes and obesity.^113 114^ The cross-sectional nature of most IBD datasets tends to limit their usefulness in inferring causal mechanisms.

Missing data are also a key challenge since these leave researchers with a choice of having to leave out particular samples or imputing missing data points, which results in reduced data and unintended errors, respectively. Also unbalanced distribution of clinical or phenotypical heterogeneity is a real-world issue affecting the interpretation of any integrative analyses. There is also a dearth of omics datasets such as proteomics, which are closer to phenotypical manifestations than other data types such as genotyping or transcriptomics. The availability of already assembled large IBD cohorts with stored biomaterial throws open multiple opportunities for improving and delivering on the research front. Sampling the biomaterials for generating the missing datatypes provides new opportunities to explore complete datasets. Thus, coordination between lead researchers and funding agencies to generate coherent multilayered datasets from the same patient samples is a major requirement. Harmonised collection, storage and usage of patient metadata and medical records are also a key challenge for inferring knowledge and clinical translation.

The contribution of disease complexity to the usefulness of multiomics datasets also extends to the composition and completeness of these datasets. The specific roles of distinct cellular populations and lineages in driving and contributing to specific phenotypes are becoming increasingly clear in IBD.^115–118^ Adding to the complexity is the recently discovered fact that mutations occur in a cell type-specific manner.^119^ Most of the datasets from organised cohorts have either profiled expression and genotyping from bulk RNA and DNA extracted from biopsy material or whole blood respectively, making it difficult to investigate the role of specific cell types in the aetiology and pathogenesis of IBD. As a case in point, Smillie *et al* demonstrated the power of profiling the expression of more than 50 cell types to pinpoint intercellular circuits which distinguish UC and healthy states.^120^

The implementation of big data and artificial intelligence approaches into clinical practice and meaningful benefits for patients is the ultimate challenge. On one hand, the deployment and operationalisation of big data are challenging, which are being addressed using computational sciences and algorithmic frameworks to manage problems related to storage, analysis, integration and interpretation of big data. Most of the infrastructures are being explored and adopted from the computer science field into healthcare. These include cloud-based data storage and analysis, and massively parallel processing hardware to tackle the rapid increase in the volumes of data from EMR, imaging and omics measurements, for example. Moreover, there is a need for user-friendly software and workflows to facilitate the integration of big data analytics into clinical practice. For instance, there have been efforts into developing NLP-based software to assist medical investigators with extracting data from plain text, such as clinical reports.^121 122^

On the other hand, many clinicians are cautious of artificial intelligence approaches mainly because most of these approaches are essentially black boxes and do not link predictions to underlying mechanisms, nor provide functional explanations for the discovered associations, correlations and recommended decisions. However, causal mechanistic insights are key for clinical applicability so as to enhance reliability and thereby patient safety, especially in a complex heterogeneous disease such as IBD. Furthermore, as poorly validated models could do more harm than good, in depth experimental and clinical validation is crucial for machine learning-based models before implementation in clinical setting. From the analytics point, interpretable machine learning models should be developed.^123^ Besides, there is a need to benchmark performance indices and parameters to evaluate the performance of machine learning techniques.^124^ Other challenges include the uncertainties associated with analyses involving the use of biological networks despite the functional context provided by the networks. Even though high-quality manually curated and benchmarked networks exist,^125 126^ analytical methods which take into account the uncertainties of individual interactions and their contextuality need to be developed. Clinical validation is fundamental for the implementation of artificial intelligence-based approaches. In one of the first randomised clinical trials using artificial intelligence, Lin *et al* compared the efficacies of childhood cataracts diagnosed by senior ophthalmologists with those from CC-Cruiser, a previously developed artificial intelligence platform for risk stratification and treatment guidance. This trial showed that regardless of the inferior accuracy of CC-Cruiser compared with senior ophthalmologists, artificial intelligence had the capacity to assist doctors in decision-making.^127 128^ All in all, clinicians are right to be sceptical of the implementation of these otherwise inexplicable approaches in clinical practice, and although there have been considerable advances in the implementation of big data, there still remain many technological, translational and cultural barriers for the assimilation of artificial intelligence approaches into clinical practice.

## Conclusion

By enabling data integration and assisting the discovery of non-trivial patterns and translatable knowledge in the integrated datasets, machine learning and systems biology offer unique opportunities to study and investigate the aetiology of complex diseases such as IBD. Machine learning guided IBD research has great potential to accelerate the formulation of cutting-edge precision medicine applications with clinical relevance and utility. However, for the promise of machine learning to come to translational fruition, there remain many stumbling blocks. However, almost all of the challenges also come with a huge potential for discovering knowledge and translating it to IBD clinical practice. It is expected that, with the availability of large IBD initiatives such as national biobanks with stored biomaterial, datasets can be made more coherent and complete, thus filling the biological gap for systems biology and the statistical gap for machine learning to produce knowledge which is closer to clinical practice and translation.

## Search strategy

Articles were retrieved from PubMed after employing the following search criteria. Two key-word groups were created, with the first one comprising “Inflammatory Bowel Disease”, “Crohn’s disease” and “ulcerative colitis” and second one comprising “machine learning”, “Artificial Intelligence”, “deep learning”, “-omics”, “big data”, “systems biology”, “network biology”, “genomics”, “transcriptomics”, “GWAS”, “proteomics” and “microbiome”. Pairwise combination of keywords from the two groups was used to search for articles published until July 2019. Only articles written in English were included.
